# Retrospective Study of Malignant Cutaneous Tumors in Dog Populations in Northwest Mexico from 2019 to 2021

**DOI:** 10.3390/ani15131979

**Published:** 2025-07-05

**Authors:** Alfonso De La Mora Valle, Daniel Gómez Gómez, Enrique Trasviña Muñoz, Paulina Haro, Melissa Macias Rioseco, Gerardo Medina Basulto, Alejandra S. Moreno, Gilberto López Valencia

**Affiliations:** 1 Instituto de Investigaciones en Ciencias Veterinarias, Universidad Autónoma de Baja California, Carr. Mexicali-San Felipe Km 3.5, Laguna Campestre, Mexicali 21386, Mexico; mora@uabc.edu.mx (A.D.L.M.V.); gomez.sergio@uabc.edu.mx (D.G.G.); etrasvina@uabc.edu.mx (E.T.M.); paulina.haro@uabc.edu.mx (P.H.); gerardom@uabc.edu.mx (G.M.B.); amoreno78@uabc.edu.mx (A.S.M.); 2 California Animal Health and Food Safety Laboratory System, University of California, Davis, 18760 Rd 112, Tulare, CA 93274, USA; mmaciasrioseco@ucdavis.edu

**Keywords:** dog, skin, cutaneous, cancer, canine tumors, neoplasia, retrospective analysis

## Abstract

Cutaneous neoplasia is among the most common illnesses in dogs and can pose significant risks. Accurate morphological diagnosis of these conditions is vital for effective treatment and management. In this retrospective study, a total of 3746 canine skin biopsies were submitted to a veterinary reference diagnostic laboratory and evaluated using histopathology. The variables assessed included age, sex, breed, lesion, location, and histopathological diagnosis. Non-neoplastic lesions accounted for 61% of all analyzed samples, while neoplastic tumors accounted for 39%. When looking at age, dogs ranging 3–6 years and 7–9 years had at least six times higher risk of developing malignant neoplasia compared to those aged 0–2 years. Among the malignant neoplasms, mast cell tumors, hemangiosarcoma, and squamous cell carcinoma were the most observed, representing 30%, 18%, and 12% of cases, respectively. The breeds most frequently affected by malignant neoplasms included Pit Bull Terriers, Boxers, and mixed breeds, all of which comprised the majority of mast cell tumor cases at 50.54%. These findings are novel in this field and may assist small animal veterinarians in making preliminary diagnoses, while also helping pet owners understand the importance of skin cancer and its early detection.

## Simple Summary

Canine cancer is among the main causes of dog fatalities worldwide. Our knowledge of its causes, biologic behavior, clinical presentation, epidemiology, and diagnostic methods are crucial for prevention and treatment. Among the several types of cancer in dogs, those affecting the skin are the most frequently diagnosed; some of these can even metastasize to peripheral lymph nodes, muscles, bone, and internal organs. Although most tumors are diagnosed in adult and elderly dogs, these have been reported in earlier life stages. Common examples of the most frequent and malignant tumors include mast cell tumors, hemangiosarcoma, squamous cell carcinoma, and malignant melanoma. These types of cancer cause considerable damage to the skin, and when metastasis occurs, a cascade of systemic reactions affects all patients. Diagnostic procedures and follow-up may involve several hospital visits and require multiple laboratory tests. Published information for veterinarians, veterinary technicians, and pet owners should be regularly updated and readily accessible to all.

## 1. Introduction

Dogs are susceptible to a wide range of diseases, including cancer. Cancer is a pathological condition characterized by dysregulated cell proliferation, resistance to apoptosis, and unchecked growth that can lead to the displacement and functional impairment of normal tissue [1]. Skin cancer is one of the most frequently diagnosed cancers in dogs [2], even 80 years ago [3]. The reason for its frequent diagnosis is its exposure to ultraviolet (UV) light and other factors [4] and because it is visually detected by owners [5]. Those with epithelial origin include squamous cell carcinomas (SCCs), adenocarcinomas, and other carcinomas. Other commonly reported neoplasms include soft tissue sarcomas, hemangiosarcoma, cutaneous lymphoma, mast cell tumor (MCT), histiocytic tumors, and malignant melanomas [6]. Precise tumor diagnosis generally requires multiple techniques, yet histopathology and cytology are still fundamental, complementary methods. Cytology presents a more affordable, less invasive sampling option and rapid turnaround times, whereas histopathology delivers more comprehensive insights by examining tissue structure [7,8].

Canine skin cancer has a worldwide distribution but is most prevalent in regions with high UV index [9,10]. They represent 37.1% of all canine skin tumors diagnosed in Northern Europe [11] and 62.5% of those in Central America [12]. Tumors related to UV radiation were the most common diagnosed in Northwest and Central Mexico, at 32.5% and 15.1%, respectively [9,10]. To date, no information has been made available about the prevalence of cutaneous tumors in dog populations in Northwest Mexico. Therefore, the aim of this study was to analyze a reference veterinary diagnostic laboratory database to determine the prevalence and associated risk factors for the most common malignant skin tumors in the dog population of Baja California, Mexico, for the period of 2019 to 2022.

## 2. Materials and Methods

### 2.1. Study Population

Between January 2019 and December 2022, the pathology laboratory at the Instituto de Investigaciones en Ciencias Veterinarias at the Universidad Autónoma de Baja California processed a total of 3746 canine skin biopsies cases for analysis. The clinical information for each case regarding breed, age, sex and anatomical location was collected from the biopsy reports assessed in this study. Dogs that did not conform to the standards of a specific breed were categorized as a mixed breed; cases missing multiple variables were excluded from the study. Tumors were categorized as either malignant or benign based on the international histological classification of tumors in domestic animals [13]; in cases where multiple tumors were present, each tumor was documented as an individual diagnosis. Instances that showed several masses with the same diagnosis were classified as disseminated and noted accordingly in the anatomical location field; only one tumor was acknowledged per patient.

This retrospective study analyzed a total of 923 biopsy cases of malignant tumors. The anatomical sites were categorized as forelimb, hindlimb, abdomen, thorax, head, neck, perineum, disseminated, and unspecified. The classification of “disseminated” was used when a specific type of tumor appeared in several skin regions.

### 2.2. Statistical Analysis

Descriptive statistics were gathered about the types of lesions identified (non-neoplastic lesions, malignant tumors, and benign tumors), as well as age, sex, breed, and the anatomical areas predominantly affected by malignant tumors. The classification and analytical approach were based on a prior study [3]. Pearson’s χ^2^ test was used to evaluate the univariable associations between each factor (sex, age, breed, and brachycephalism) and tumor type (malignant vs. benign). The factors examined were sex (female, spayed female, male, and neutered male; reference category = neutered male), breed (reference category = mixed breed), and age (reference category = dogs under 2 years old). Dogs that were 2 years old served as the reference group as they represented the youngest age group with enough cases for comparison with other age groups.

Subsequently, a multivariable logistic regression analysis was conducted to assess the combined effects of age, sex and breed on the likelihood of developing a malignant tumor. The results were expressed as odds ratios (ORs) along with their corresponding 95% confidence intervals (CIs), and *p* < 0.05 was deemed significant. All statistical analyses were performed using STATISTIX^®^ version 9.1.

### 2.3. Graphic Illustration

Graphical Abstract: Created in BioRender. Moreno, A. (2025) https://BioRender.com/l6jkrqj.

## 3. Results

A total of 3746 skin biopsies were analyzed over the period of January 2019 to December 2021, which included submissions from the major cities of Baja California. Of these biopsies, 2283 (60.9%) were classified as non-neoplastic lesions (follicular cysts, acute or chronic inflammation, etc.), and 1463 (39%) were neoplastic tumors (Table 1). Most of the neoplastic tumors were classified as malignant (923, 63%), and 540 were benign (36.9%). We organized the results from malignant tumors into four categories of age and four categories of sex. Malignant skin tumors were found in dogs over 7 years old (76.9%) with a median age of 9 years old. The rates were similar between males and females (516 males and 404 females).

**Figure 1:**
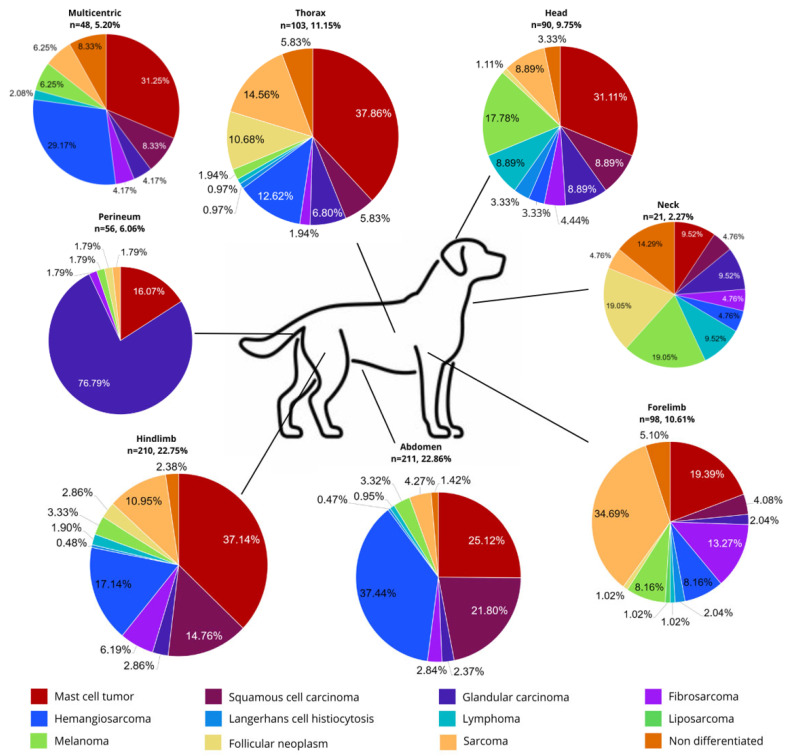
Most common anatomic regions with the presence of the twelve more common malignant tumors in dogs (n = number of tumors) and relative frequency (%).

Table 2 summarizes the most common malignant skin tumors. MCTs, hemangiosarcoma, SCC, soft tissue sarcoma, and glandular carcinoma were the most frequently diagnosed. MCTs occurred most often with 277 cases (30%), of which 227 (81.9%) were diagnosed as grade 1, 42 (15.1%) as grade 2, and 8 (2.8%) as grade 3 based on the Patnaik Grading System [14], and 269 (97.1%) were diagnosed as low-grade and 8 (2.8%) as high-grade based on the Kiupel Grading System [15], followed by hemangiosarcoma with 167 cases (18%) and SCC with 112 cases (12.1%). Glandular carcinoma was the fifth most common tumor type with 77 (8.3%) cases, and this study consisted of 37 (48%) hepatoid adenocarcinomas; 34 (44.1%) apocrine adenocarcinomas, of which 26 (76.4%) were cutaneous and 8 (23.5%) were from anal sac origin; 3 (3.8%) sebaceous; 2 (2.5%) ceruminous; and 1 (1.2%) undifferentiated. Figure 1 shows the main anatomical distribution of these tumors by region. Most MCTs were found on the hind limb, the abdomen, and the thorax. Hemangiosarcoma and SCC had similar patterns of distribution on the hind limb and abdomen. Melanoma and cutaneous lymphoma were particularly frequent on the head (16% and 40%, respectively).

The most frequently affected breeds included Pit Bull Terriers, mixed breeds, Boxers, Labrador Retrievers, Schnauzers, and Bulldogs (Table 3). Pit Bull Terriers were the most frequently diagnosed with malignant skin tumors (224 (24%) of the total of 923 cases reported), followed by Boxers and Labrador Retrievers. In these breeds, the most common malignant tumors varied in frequency. Pit Bull Terriers had particularly high frequencies of MCTs, hemangiosarcoma, and SCC, as did Boxers. In Labrador Retrievers, MCTs were also the main malignant tumor, but other malignant tumors had a similar frequency for this breed. A large number of cases were classified as mixed breeds, which had a high frequency of MCTs, hemangiosarcomas, and sarcomas. This group may have included breeds similar to Pit Bull Terriers (at least in part).

A univariable analysis was conducted to examine the relationship between malignant tumors and various factors (Table 4). No association was found concerning sex, but age showed a significant association. Dogs 2 years old or younger had the lower risk of developing malignant tumors in comparison to dogs between the ages of 3–6 years, who were found to have seven times higher risk of developing malignant tumors, followed by those aged 7–9 years and those over the age of 10.

A comparison was carried out between mixed breed and purebred dogs, which revealed that Pit Bull Terriers had the highest risk, which was six times higher, followed by Boxers, Chihuahuas, and Labrador Retrievers. Brachycephalic breeds had 2.84 times greater risk of developing malignant tumors. Table 5 presents the results of the multivariable logistic regression analysis, which illustrate the relationship between malignant and benign tumors and the factors of sex, age, and breed. In both the univariable and multivariable analyses, the association between age and breed with malignant tumors was consistent (Table 5), with 1.01 times higher risk with respect to breed and 1.41 times higher risk with respect to age.

## 4. Discussion

In this study, we examined 3746 skin biopsies in terms of age, breed, sex, and reproductive status to enhance our understanding of risk factors associated with several types of skin cancer in dogs residing in Mexicali, Baja California, Mexico. The findings indicate that 39.0% (1463/3746) of the skin biopsies analyzed were neoplastic. This is a lower percentage than that reported in studies conducted in tropical regions of Brazil, where Santos et al. (2020) [16] documented a rate of 59.2% (1266/2138), and Machado et al. (2018) [17] indicated a rate of 85.7% (468/617) involving neoplasms in skin lesions [16,17]. Murakatirwa et al. (2005) [18] noted a figure of 60.0% (540/900) in Zimbabwe, while reports from temperate areas of Serbia showed rates as high as 81.6% (1984/2432) [18,19].

In two studies conducted in Central Mexico, Fajardo et al. (2013) [20] and García et al. (2019) [10] documented rates of 52.9% (91/172) and 59% (231/393), respectively, and stipulated that tumors associated with exposure to solar radiation were the most frequently reported [10,20]. The discrepancy between the present study and previous studies may be attributed to the extreme climate in Mexicali throughout the year, which has temperatures ranging from 2 to 44 °C [21]. As a result, many pets receiving treatment in veterinary clinics (the studied population) are often shielded from harsh weather and thus from direct sunlight and UV exposure.

Signs of malignancy occurred in 63% (923/1463) of the total neoplasms, which is comparable to the figures of 62.5% (493/789) reported by Medina et al. (2017) [12] in Peru and 56.7% (813/1435) noted by Kok et al. (2019) [22] in Japan. However, this percentage is significantly higher than the rate of 21.0% (160/765) malignant neoplasms identified by Brønden et al. (2010) [23] in Denmark. Several reports, all histopathology based, indicate rates of malignant neoplasms ranging from 33% to 48%; in Central Mexico, rates are consistent. García et al. (2019) [10] reported that out of 393 skin biopsies, 156 were malignant, representing 39.7%, while Fajardo et al (2013) [20]. reported 33% of malignant results out of 91 skin biopsies, similarly to what was reported in Poland and Northern Portugal at 34.98% and 37.1%, respectively. Slightly higher figures were reported in Serbia and Brazil with 47.73% and 45.94%, respectively. However, these studies were conducted in various regions with differing climates and urban characteristics, making it challenging to pinpoint a specific reason for the discrepancies in proportions among neoplasms. Kok et al. (2019) [22] hypothesizes that benign neoplasms might be underdiagnosed due to a lack of surgical resections or histopathological evaluations because of their assumed benign nature, which we acknowledge as a plausible yet undesirable explanation.

We divided ages into groups: ≤2 years, 3–6 years, 7–9 years, and >10 years. Compared to those under 2 years old, the likelihood of developing malignant neoplasms was over six times greater in each age group. However, this likelihood did not increase with age; instead, it remained consistent and even diminished in dogs older than 10 years. This observation may be attributed to the fact that the population of dogs under 2 years was between 100 and 200 times smaller than that of the older groups.

Additionally, Smiech et al. (2023) [2] noted dogs aged 0–3 years had a four times lower probability of malignant neoplasms, suggesting that the rate might be overestimated due to the high incidence of histiocytomas in dogs younger than 5 years. However, this consideration does not apply to our study as we excluded benign tumors from our analysis. We also identified other research that supports the notion of increased frequency in older dogs [11,22,24], although these studies do not distinguish between overall neoplasia occurrence and malignant neoplasia specifically.

In general, we found that intact males and spayed females had a 1.32 and 1.37 times higher likelihood of developing malignant neoplasia compared to neutered males, respectively. Intact females did not exhibit a higher likelihood of developing skin neoplasia. However, in the multivariable analysis, sex was not determined to be a risk factor. In contrast, Smiech et al. (2023) [2] and Graf et al. (2018) [24] reported that females had a 1.21 and 1.075 times greater likelihood of malignant neoplasia than males, while Machado et al. (2018) [17] found no significant difference between sexes.

The breed that had the highest rate of malignant skin neoplasms was Pit Bull Terriers, followed by mixed breeds, Boxers, and Labrador Retrievers. This can vary significantly across different studies due to differing breed populations in each location. Notably, Denis et al. (2020) [25] noted that in Uruguay, four out of the five most prevalent breeds were consistent with those in our study. Unfortunately, that research focused solely on MCTs, although they were the most frequently observed neoplasms in our findings.

The three most prevalent neoplasms among the most frequent nine were MCTs (30%), hemangiosarcoma (18%), and SCC (12%). These neoplasms frequently appear in various studies [7,10,16,19,22,24]. As in the present study, both Santos et al. (2020) [16] and Martins et al. (2022) [11] identified the same neoplasms as the three most common. However, Martins et al. (2020) [11] reported a prevalence of MCTs that was nearly double our figure (61.1%). Two studies indicate even higher prevalence rates for MCTs than the figures noted by Martins et al. (2022) [11]. For instance, Rodríguez et al. (2023) [26] reported a 67% prevalence, but this statistic only pertained to three malignant cutaneous round cell neoplasms. Conversely, Brønden et al. (2010) indicated a 71.2% prevalence of MCTs in malignant cutaneous neoplasms, although this study also incorporated cytological diagnoses alongside histopathology [23].

In terms of breeds, Pit Bull Terriers were the most affected by MCTs, ranking just below the group of less frequent breeds. Santos et al. (2020) [16] and Kok et al. (2019) [22] identified mixed breeds as the most affected. Regarding sex, our findings align closely with those of Santos et al. (2020) [16] and Kok et al. (2029) [22], who noted a marginally higher prevalence in females (between 1:1.22 and 1:1.30). The average age of occurrence was 8.5 years, which is similar to the findings of Santos et al. (2020) [16]. The abdomen was the primary site for MCTs, which correlates with other reports [7,16,23,24,25,27]. However, Santos et al. (2020) [16] identified the scrotum as the main location. Among all studies, only that of Santos et al. (2020) [16] indicated a frequency greater than 50% (54.2%), while other studies showed frequencies between 24% and 40% for hind limbs and between 18% and 42% for the trunk.

Among the most relevant risk factors for cutaneous hemangiosarcomas and squamous cell carcinomas is excess ultraviolet radiation (UVR) exposure [28]. The UV index in Baja California varies throughout the year, ranging from moderate to extreme according to the Global Solar UV Index (UVI) from the World Health Organization (WHO), reaching extreme levels in the summer months (12–13), very high to high levels in the spring and fall (6–9), and high to moderate levels in winter (6–7) [29,30]. Hemangiosarcomas exhibit varying prevalence rates across different studies, ranging from 1.79% [19] to 24.3% [16]. In our investigation, the most frequently impacted breed was Pit Bull Terriers, which is consistent with the findings of Santos et al. (2020) [16]. Concerning age, we determined a mean age of 8.5 years for hemangiosarcoma diagnosis, which is close to the finding of 8.3 years by Santos et al. (2020) [16]. However, our study revealed a male–female ratio of 1:1.50, while Santos et al. (2020) [16] reported a slightly higher ratio of 1:0.98 [16]. In both studies, the predominant site of occurrence was the abdomen with rates of 37.44% and 28.5%, respectively. However, Kok et al. (2019) [22] identified the forelimb as the most common site, albeit at a significantly lower rate of 4.35%.

For SCC, both our study and that by Santos et al. (2020) [16] found that Pit Bull Terriers were the most affected breed, while Silva-Hidalgo et al. (2015) [9] identified Bull Terriers and Kok et al. (2019) [22] found mixed breeds to be the most affected breeds. The average age of SCC occurrence in our research and that reported by Santos et al. (2020) [16] were nearly identical (8.4 and 8.3 years, respectively). Again, there was variation in sex. Both our study and that by Kok et al. (2019) [22] demonstrated a higher prevalence in females (male–female ratios of 1:1.39 and 1:1.91, respectively), although the discrepancies are considerable. However, Santos et al. (2010) [16] and Silva-Hidalgo et al. (2015) [9] reported a greater prevalence of SCC in males (male–female ratios of 1.01:1 and 1:1.62, respectively), but again, the differences are quite significant. In terms of the most frequent site for SCC presentation, we found the abdomen to be the primary location at 21.80%, while Santos et al. (2020) [16] reported a rate of 16.8%. Kok et al. (2019) [22] reported a 21.02% occurrence in the forelimb, whereas Silva-Hidalgo et al. (2015) [9] observed 33.0% in the foreskin.

In terms of breed predisposition, our findings indicate that the Pit Bull Terriers most frequently had neoplasia and showed considerable variance when compared to mixed breeds. However, this discrepancy might be influenced by the number of Pit Bull Terriers included in our study. According to Denis et al. (2020) [25], Pit Bull Terriers were the fourth most prevalent breed associated with malignant skin neoplasia. Conversely, even though we recorded significantly fewer cases involving Boxers, we still observed a notable difference concerning their heightened likelihood of developing malignant skin neoplasms.

Similarly, a stronger association was found in brachycephalic breeds, such as Boxers. Nonetheless, a UK study examining veterinary records of 3219 Boxers did not reveal any statistical differences in susceptibility to skin nodules or neoplasms leading to death. However, that study identified a higher tendency for females (*p* = 0.006) to develop skin nodules [31]. In the case of brachycephalic breeds, UK research indicated that brachycephalic breeds had a protective advantage against developing skin nodules compared to dogs with different skull shapes (OR = 0.60, *p* < 0.001). However, the sample size of brachycephalic dogs was significantly smaller (57 dogs) compared to non-brachycephalic dogs (406 dogs). Even when performing a multivariable analysis that accounted for factors like skull shape, age, sex, and weight, no significant statistical differences were identified (*p* = 0.972) [32].

This study has limitations inherent to its retrospective design, in this context, soft tissue sarcomas, and the use of markers will be desired to have a better approach in these groups. Multicenter studies with advanced diagnostic tools are required for better characterization, particularly in those malignant neoplasms with poor prognosis, this can provide useful data for clinicians and owners.

Future studies of canine cancer should always consider the use and implementation of a canine tumor registry using a standardized classification system such as Vet-ICD-O (Veterinary adaptation of the International Classification of Diseases for Oncology). This standardization allows consistent diagnosis reporting across institutions and geographic regions and enables the early identification of emerging cancer trends in canine populations [33,34].

## 5. Conclusions

This study on cutaneous tumors shares several similarities with previous publications and shows consistency with the prevalence of the main tumor malignancies affecting dogs. It is interesting that only a small number of malignancies were diagnosed, and most of them were found in dogs over 7 years of age. Among the tumors with high incidence, SCC and hemangiosarcoma were particularly frequent in Pit Bull Terriers and mixed breeds with similar phenotypes, and most of these tumors affected the ventral abdomen and inguinal areas. Our findings may inform future research and clinical practices aimed at diagnosing and preventing skin tumors, while also serving as a valuable tool for educating pet owners about their implications for animal health.

## Figures and Tables

**Table 1:** Characteristics of the 3746 cases from 2019 to 2021.

Analyzed Samples		3746 (%)
Prevalence of non-neoplastic lesions		2283/3746 (60.9)
Prevalence of neoplastic tumors		1463/3746 (39)
Neoplastic tumor samples		1463 (%)
Prevalence of malignant tumors		923/1463 (63)
Prevalence of benign tumors		540/1463 (36.9)
Frequency of malignant tumors by age and gender		
Characteristic	Category	N (%)
Age	≤2 years	21 (2.27)
	3–6 years	191 (20.6)
	7–9 years	333 (36)
	>10 years	378 (40.9)
Median(range)		9.0 (2.0–18.0)
Sex *	Male	356 (38.5)
	Neutered male	160 (17.3)
	Female	207 (22.4)
	Spayed female	197 (21.3)

* In three cases, the sex was not specified.

**Table 2:** Most common skin malignant tumors. We show the total cases for each tumor, the male to female ratio, the median and average age, and the three most common regions for each tumor.

Tumor	Cases (%)	Male–Female Ratio	Median (Age)	Average (Age)	Region * n (%)
Mast cell tumor	277 (30)	1:1.06	8	8.36	HL: 78 (28.1) A: 53 (19.1) T: 39 (14)
Hemangiosarcoma	167 (18)	1:1.49	8	8.58	A: 79 (47.3) HL: 36 (21.5) T: 13 (7.7)
Squamous cell carcinoma	112 (12.1)	1:1.36	8	8.4	A: 46 (41) HL: 31 (27.6) H: 8 (7.1)
Sarcoma	101 (10.9)	1:1.02	10	9.84	FL: 34 (33.7) HL: 23 (22.8) T: 15 (14.8)
Glandular carcinoma	77 (8.3)	1:1.96	11	9.98	P: 43 (55.8) H: 8 (10.3) T: 7 (9)
Melanoma	52 (5.6)	1:1	10	10.13	H: 16 (18.1) A: 7 (12.5) HL: 7 (12.5)
Fibrosarcoma	47 (5)	1:1.76	8	8.15	FL: 13 (30.7) HL: 13 (30.7) A: 6 (12.7)
Undifferentiated	33 (3.5)	1:1.6	8.5	8.7	T: 6 (18.1) FL: 5 (15.1) HL: 5 (15.1)
Lymphoma	20 (2.1)	1:1.22	7	7.7	H: 8 (40) HL: 4 (20) A: 2 (10)
Other **	37 (4)	0.68:1	9	8.6	
Total	923 (100)	1:1.2			

* A: abdomen; FL: forelimb; HL: hindlimb; H: head; T: thorax; P: perineum. ** Other: follicular tumors, histiocytosis, liposarcoma.

**Table 3:** Frequency of malignant tumors among dog breeds from 2019 to 2021; numbers in parenthesis represent the percentage of the type of neoplasm by breed.

Breed	MCT *	Hem	SCC	Sar	Gl. Car	Und	Mel	Fib	Other	Total
Pitbull Terrier	61 (27.2)	88 (39.2)	42 (18.7)	10 (4.4)	3 (1.3)	7 (3.1)	5 (2.2)	6 (2.6)	2 (0.8)	224 (100%)
Mixed Breeds	42 (27.8)	20 (13.2)	14 (9.2)	24 (15.8)	17 (11.2)	7 (4.6)	11 (7.2)	7 (4.6)	9 (5.9)	151 (100%)
Boxer	37 (47.4)	14 (17.9)	13 (16.6)	3 (3.8)	1 (1.2)	3 (3.8)	2 (2.5)	3 (3.8)	2 (2.5)	78 (100%)
Labrador Retriever	16 (34.7)	2 (4.3)	4 (8.6)	7 (15.2)	5 (10.8)	1 (2.1)	3 (6.5)	5 (10.8)	3 (6.5)	46 (100%)
Schnauzer	9 (25)	3 (8.3)	4 (11.1)	7 (19.4)	2 (5.5)	1 (2.7)	7 (19.4)	1 (2.7)	2 (5.5)	36 (100%)
Bulldog	17 (53.1)	5 (15.6)	5 (15.6)	3 (9.3)	0 (0.0)	1 (3.1)	0 (0.0)	1 (3.1)	0 (0.0)	32 (100%)
Other	95 (26.6)	35 (9.8)	30 (8.4)	47 (13.2)	49 (13.7)	13 (3.6)	24 (6.7)	24 (6.7)	39 (10.9)	356 (100%)
Total	277	167	112	101	77	33	52	47	57	923 (100%)

* MCT: mast cell tumor; Hem: hemangiosarcoma; SCC: squamous cell carcinoma; Sar: sarcoma; Gl. Car: glandular carcinoma; Und: undifferentiated; Mel: melanoma; Fib: fibrosarcoma.

**Table 4:** Pearson’s χ^2^ test analysis shows the association between malignant vs. benign tumors with sex, age, breed, and brachycephalism.

Factor	Malignant n (%)	Benign n (%)	Odds Ratio	95% IC	*p* Value
Sex					
Neutered male	160 (58.6%)	113 (41.3%)	1	Ref	Ref
Male	356 (66.5%)	179 (33.4%)	1.32	0.98–1.79	0.06
Female	207 (58.8%)	145 (41.1%)	0.95	0.69–1.31	0.77
Spayed female	197 (66.1%)	101 (33.8)	1.37	0.98–1.93	0.06
Age (years)					
≤2	21 (22.8%)	71 (77.1%)	1	Ref	Ref
3–6	191 (67.9%)	90 (32%)	7.17	4.19–12.4	0.0001
7–9	333 (64.7%)	181 (35.2%)	6.22	3.70–10.45	0.0001
>10	378 (66.4%)	191 (33.5%)	6.69	3.98–11.22	0.0001
Breed *					
Mixed	151 (52.7%)	135 (47.2%)	1	Ref	Ref
Pitbull	224 (87.8%)	31 (12.1%)	6.46	4.15–10.04	0.0001
Boxer	78 (82.1%)	17 (17.8%)	4.10	2.31–7.28	0.0001
Labrador	46 (67.6%)	22 (32.3%)	1.86	1.06–3.26	0.02
Chihuahua	72 (67.9%)	34 (32%)	1.89	1.18–3.02	0.007
Brachycephalic					
No	532 (55.3%)	429 (44.6%)	1	Ref	Ref
Yes	391 (77.8%)	111 (22.1%)	2.84	2.22–3.63	0.0001

* A total of 63 breeds were evaluated, including one category of mixed breeds, and only the breeds with significant differences were added in the table.

**Table 5:** Multivariable logistic regression analysis shows the association between malignant vs. benign tumors with sex, age, and breed.

Factor	Malignant n (%)	Benign n (%)	Odds Ratio	95% IC	*p* Value
Sex					
Male	356 (66.5%)	179 (33.4%)	1.04	0.95–1.14	0.29
Spayed female	197 (66.1%)	101 (33.8%)			
Female	207 (58.8%)	145 (41.1%)			
Neutered male	160 (58.6%)	113 (41.3%)			
Age (years)					
3–6	191 (67.9%)	90 (32%)	1.43	1.25–1.62	0.0001
>10	378 (66.4%)	191 (33.5%)			
7–9	333 (64.7%)	181 (35.2%)			
≤2	21 (22.8%)	71 (77.1%)			
Breed *					
Pitbull	224 (87.8%)	31 (12.1%)	1.02	1.01–1.03	0.0001
Boxer	78 (82.1%)	17 (17.8%)			
Chihuahua	72 (67.9%)	34 (32%)			
Labrador	46 (67.6%)	22 (32.3%)			
Bulldog	32 (61.5%)	20 (38.4%)			
Mixed	151 (52.7%)	135 (47.2%)			

* A total of 63 breeds were evaluated, including one category of mixed dogs, and only the breeds with the highest frequency of cancer cases are presented in this table. The categories of each variable are arranged in order of frequency.

## Data Availability

The original contributions presented in this study are included in the article. Further inquiries can be directed at the corresponding author.

## Abbreviations

The following abbreviations are used in this manuscript:

MDPI	Multidisciplinary Digital Publishing Institute;
DOAJ	Directory of open access journals;
TLA	Three-letter acronyms;
LD	Linear dichroism;
UV	Ultraviolet light;
UVR	Ultraviolet radiation;
UVI	Global Solar UV Index;
WHO	World Health Organization;
SCCs	Squamous cell carcinomas;
MCTs	Mast cell tumors.
